# Rodent phylogeny revised: analysis of six nuclear genes from all major rodent clades

**DOI:** 10.1186/1471-2148-9-71

**Published:** 2009-04-02

**Authors:** Shani Blanga-Kanfi, Hector Miranda, Osnat Penn, Tal Pupko, Ronald W DeBry, Dorothée Huchon

**Affiliations:** 1 Department of Zoology, George S. Wise Faculty of Life Sciences, Tel-Aviv University, Tel-Aviv 69978, Israel; 2 Department of Biological Sciences, University of Cincinnati, Box 210006, Cincinnati, Ohio, 45221-0006, USA; 3 Department of Cell Research and Immunology, George S Wise Faculty of Life Sciences, Tel-Aviv University, Tel-Aviv 69978, Israel; 4 Department of Biology, Texas Southern University, 3100 Cleburne Street, Houston, TX 77004, USA

## Abstract

**Background:**

Rodentia is the most diverse order of placental mammals, with extant rodent species representing about half of all placental diversity. In spite of many morphological and molecular studies, the family-level relationships among rodents and the location of the rodent root are still debated. Although various datasets have already been analyzed to solve rodent phylogeny at the family level, these are difficult to combine because they involve different taxa and genes.

**Results:**

We present here the largest protein-coding dataset used to study rodent relationships. It comprises six nuclear genes, 41 rodent species, and eight outgroups. Our phylogenetic reconstructions strongly support the division of Rodentia into three clades: (1) a "squirrel-related clade", (2) a "mouse-related clade", and (3) Ctenohystrica. Almost all evolutionary relationships within these clades are also highly supported. The primary remaining uncertainty is the position of the root. The application of various models and techniques aimed to remove non-phylogenetic signal was unable to solve the basal rodent trifurcation.

**Conclusion:**

Sequencing and analyzing a large sequence dataset enabled us to resolve most of the evolutionary relationships among Rodentia. Our findings suggest that the uncertainty regarding the position of the rodent root reflects the rapid rodent radiation that occurred in the Paleocene rather than the presence of conflicting phylogenetic and non-phylogenetic signals in the dataset.

## Background

The order Rodentia is the most diverse among placental mammals: extant rodent species represent half of the placental diversity (2,277 species divided into 33 families) [1]. Morphological phylogenetic approaches have identified characters supporting a common origin (monophyly) of rodents, and clustered rodents and lagomorphs (rabbits, pikas) in a clade called Glires [2]. Morphological studies also generally agree on the number and content of rodent families [1,3,4]. However, the description of the relationships among rodent families has been confounded by rampant convergent evolution of morphological characters [5]. Based on morphological characters, rodents have been divided into either two or three suborders. The first system, suggested by Brandt, divides rodents into three suborders, Myomorpha, Sciuromorpha, and Hystricomorpha, based on the position of masticatory muscles (the masseters) [6]. However, it has since been proven that this character is homoplasic and that this classification does not reflect evolutionary relationships [7,8]. The second system, proposed by Tullberg, divides rodents into two suborders, Sciurognathi and Hystricognathi, based on the position of the incisors and the angle of the jaw [9]. The monophyly of Hystricognathi has been accepted, based on the identification of additional morphological synapomorphies, but the Sciurognathi are usually considered to be paraphyletic [10]. Debates on the relationships within Sciurognathi and their relationships with Hystricognathi are the subject of numerous morphological papers [reviewed in [11]]. Molecular studies were expected to clarify the relationships among rodents. However, early studies based on molecular data complicated the understanding of rodent evolution by suggesting that rodents are paraphyletic [12-14]. These results initiated lively debates concerning evolutionary relationships among rodents and their place among placental mammals [15-17]. Phylogenetic conclusions supporting rodent paraphyly have been criticized, because they were based on a very limited taxonomic sampling. It has been suggested that increasing the sampling of rodent diversity [2] and/or mammalian diversity [18] would have supported rodent monophyly. Additionally, over-simplified models have been shown to erroneously support rodent paraphyly [19]. Recent analyses based on a representative sampling of rodent taxonomic diversity and using model-based methods of sequence analysis have strongly supported the monophyly of rodents [20-24].

Within Rodentia, molecular analyses suggest that rodents are divided into seven well-supported clades: 1-Anomaluromorpha (scaly-tailed flying squirrels, springhares), 2-Castoridae (beavers), 3-Ctenohystrica (gundi, porcupines, guinea-pigs), 4-Geomyoidea (pocket gophers, pocket mice), 5-Gliridae (dormice), 6-Myodonta (rats, mice, jerboas), and 7-Sciuroidea (mountain beavers, squirrels, woodchucks) [25-28]. However, several evolutionary relationships within Rodentia are still debated. Recent studies have suggested that these seven clades are clustered into three main lineages: 1 – Anomaluromorpha, Castoridae, Geomyoidea, and Myodonta together form the "mouse-related clade"; 2 – Sciuriodea and Gliridae form the "squirrel-related clade"; and 3 – Ctenohystrica forms the third lineage [29-32]. However, most studies have not been able to solve the relationships among these three clades. Recently, Montgelard et al. [32] analyzed mitochondrial genes as well as nuclear exonic and intronic sequences, and found significant support in favor of a basal position of the "mouse-related clade". This result was dependent on the removal of the fastest evolving characters from the dataset, suggesting that mutational saturation might explain the inconclusive placement of the rodent root.

More generally, Rodriguez-Ezpeleta et al. [33] have shown that weakly supported nodes can sometimes be explained by the presence of conflicting phylogenetic and non-phylogenetic signal in a dataset. Three methods to reduce the non-phylogenetic information have been suggested: identification and removal of fast-evolving positions, character-recoding (e.g., RY coding), and the use of a site-heterogenous mixture model (e.g., CAT) [34].

Here, we aimed to resolve rodent relationships at the family level and above. We established a comprehensive dataset including six nuclear gene fragments from 41 rodent species together with eight outgroup species. We were able to solve most evolutionary relationships among rodent families. In order to minimize conflicting signals and thus solve the debated basal rodent relationships, we applied the three methods suggested by Rodriguez-Ezpeleta et al. [33]. We show that none of these methods, nor the use of more complex evolutionary models, can significantly solve basal rodent relationships. Additionally, some of our analyses, surprisingly, suggest a basal position of the squirrel-related clade and significantly reject the basal position of the "mouse-related clade" supported by Montgelard et al. [32]. We thus propose that the lack of resolution at the base of the rodent tree may reflect rapid rodent radiation, rather than conflicting phylogenetic signals.

## Results and discussion

### The rodent phylogeny

**Figure 1:**
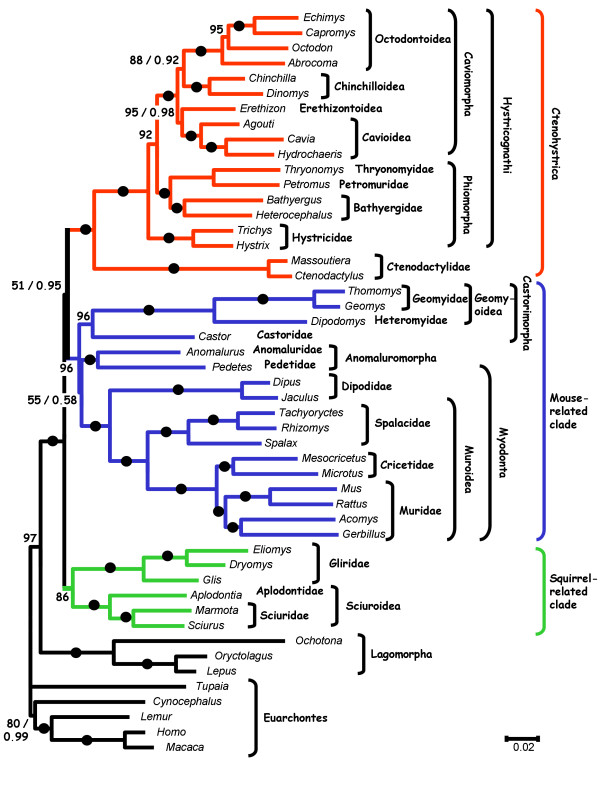
**ML tree (-ln likelihood = 85,018.88) obtained for the concatenated dataset under the GTR+Γ+I model of sequence evolution**. For each node the ML bootstrap percentage (BP) and the Bayesian posterior probabilities (PP) are given at the right and left of the slash, respectively. Branches with maximal support value (i.e., BP = 100, PP = 1.0) are indicated by black dots.

Maximum likelihood (ML) and Bayesian phylogenetic analyses, based on the combined nucleotide datasets, result in a well-resolved phylogeny (Figure 1), in agreement with the division of rodents into three major clades: the mouse-related clade (Bootstrap Percentage (BP) = 96, Posterior Probability (PP) = 1.0), the squirrel-related clade (BP = 86, PP = 1.0) and the Ctenohystrica (BP = 100, PP = 1.0).

#### The mouse-related clade

The mouse-related clade comprises three main lineages: Myodonta, Anomaluromorpha (Anomaluridae and Pedetidae), and Castorimorpha (Geomyoidea and Castoridae). The monophyly of this clade was first found in molecular studies [25,30-32] and later corroborated by a morphological analysis of extant and fossil taxa [8]. However, two recent molecular analyses cast doubt on the validity of the mouse-related clade. First, analysis of complete mitochondrial protein-coding genomes placed *Anomalurus *as a sister taxon of the Hystricognathi [24]. Second, structural analysis of B1 retroposon elements suggested that Castoridae could be an early diverging family within rodents [35]. Our analysis strongly rejects both of these possibilities. The best alternative to monophyly of the mouse-related clade is significantly less likely than the ML tree, based on the approximately unbiased (AU) test (Table 1, *p*-value = 0.02). Analyses using RY coding or removal of third-codon positions, as well as partitioned analyses strongly support the monophyly of the mouse-related clade (Table 2, BP = 94–100). When protein sequences are analyzed, the monophyly of the mouse-related clade is still supported, albeit with lower bootstrap support (BP = 77). It is likely that the disagreement between our analysis and that of Horner et al. [24] stems from the fact that the latter was based on only six non-muroid species. With regard to the B1 retroposon study, the position of Castoridae presented by Veniaminova et al. [35] may be an artifact, because analysis of SINE insertion loci in rodents supports the monophyly of the mouse-related clade [36].

**Table 1:** Results of likelihood-based tests of alternative topologies

**Constraint**	**Diff -ln L**	**AU**	**KH**	**wSH**
Unconstrained (ML tree)	<79199.95>	0.855	0.537	0.999
Ctenohystrica at the base of the rodent tree	0.5	0.650	0.463	0.917
Myodonta at the base of the mouse-related clade	1.1	0.604	0.386	0.928
Mouse-related clade at the base of the rodent tree	2.5	0.391	0.296	0.853
Anomaluromorpha at the base of the mouse-related clade	3.0	0.159	0.156	0.733
[Cavioidea+Erethizontoidea] not monophyletic	7.4	0.148	0.087	0.360
[Chinchilloidea+Octodontoidea] not monophyletic	8.0	0.206	0.149	0.471
Paraphyly of the squirrel-related clade	11.9	0.123	0.085	0.293
Caviomorpha at the base of Hystricognathi	12.2	0.128	0.079	0.278
Phiomorpha at the base of Hystricognathi	12.2	0.128	0.079	0.278
Paraphyly of the mouse-related clade	31.4	**0.024**	**0.017**	**0.050**
Paraphyly of Ctenohystrica	51.8	**3e-4**	**< 1e-10**	**< 1e-10**

Diff -ln L: observed log-likelihood difference between the ML topology and the alternative. AU: *p*-values of the approximately unbiased test. KH: *p*-values of the Kishino-Hasegawa test. wSH: *p*-values of the weighted Shimodaira – Hasegawa test. Significant *p*-values are indicated in bold.

**Table 2:** Maximum likelihood bootstrap support of main rodent relationships under different coding models

	**Unpartitioned models**				**Partitioned DNA models**		
**Coding ** **model** **/Node**	All three codon positions	First two codon positions and third codon with RY coding	First two codon positions only	Protein sequences	A partition per gene (6 partitions)	A partition per codon position (3 partitions)	A partition per codon position per gene (18 partitions)

Squirrel-related clade at the base of the rodent tree	51	35	40	41	46	63	75
Mouse-related clade at the base of the rodent tree	15	28	less than 1	less than 1	17	9	15
Ctenohystrica at the base of the rodent tree	25	30	48	47	31	22	8
Monophyly of squirrel-related clade	86	90	89	98	92	92	95
Monophyly of mouse related clade	96	99	94	77	100	97	99
Monophyly of Ctenohystrica	100	100	99	100	100	100	100
[Anomaluromorpha + Myodonta] monophyly	55	54	65	37	50	72	48
Hystricidae at the base of the Hystricognathi	92	91	89	89	95	91	91

Previous phylogenetic reconstructions were unable to solve the relationships among the three main lineages of the mouse-related clade (Myodonta, Anomaluromorpha, and Castorimorpha), and all three possible evolutionary relationships have been suggested [22,25,26,28,31,32,36]. Our phylogenetic inference based on the full nucleotide dataset suggests the grouping of Anomaluromorpha with Myodonda (Figure 1). However, bootstrap and Bayesian support is at best moderate across the analyses considered (Table 2, BP = 37–72, PP = 0.58). In agreement with the bootstrap analysis, an AU test does not reject either alternative hypotheses (Table 1, *p*-value = 0.159–0.604). Additional data are thus needed to resolve the relationships at the base of the mouse-related clade. All other nodes within the mouse-related clade are well supported and alternatives are rejected based on an AU test (data not shown).

#### The squirrel-related clade

The grouping of Gliridae and Sciuridae has been recognized in morphological studies based on middle ear features [37], arterial pattern [38], and by most molecular analyses. Nevertheless, high support values have seldom been obtained to support this relationship [22,25-27,29,31,32]. This node is well supported in our study (BP = 86, PP = 1.0). It is also supported in our analyses using different coding and partitions approaches (Table 2, BP = 86–98). However, alternatives to the monophyly of this clade are not rejected according to the AU test (Table 1, *p*-value = 0.123).

#### The Ctenohystrica

The clustering of Ctenodactylidae and Hystricognathi is highly supported (BP = 100, PP = 1.0). Previous knowledge of relationships within hystricognaths has been based either on a single gene (vWF or 12sRNA) for many hystricognath species (22–23 species) [39,40] or on multiple genes (3–6 genes) for fewer species (8–13 species) [22,29,32,41]. The present dataset expands that of Huchon et al. [29] by the addition of two nuclear gene fragments and four hystricognath taxa (in particular, a second representative of the Hystricidae). This expanded dataset allows us to solve the debated relationships within Hystricognathi. We find strong support for a basal position of Hystricidae within Hystricognathi (Figure 1, Table 2, BP = 89–95, PP = 1.0), while this position was previously only weakly supported [25,26,29,40]. However, AU tests do not reject alternative positions of Hystricidae (Table 1, *p*-value = 0.128).

Phylogenetic relationships among South-American hystricognaths (i.e., Caviomorpha) have long been debated. Caviomorphs have been found to comprise four distinct lineages (Cavioidae, Chinchilloidea, Erethizontoidea, and Octodontoidea) [40]. Our results confirm that chinchilla rats (Abrocoma) are not related to *Chinchilla *but rather belong to the Octodontoidea (BP = 100, PP = 1.0) [40,42,43]. Previous molecular trees did not resolve the relationships among the four caviomorph lineages with high bootstrap support, and various alternative topologies have been suggested [22,25,26,29,40]. Our data support a sister clade relationship between Cavioidea and Erethizontoidea (BP = 95, PP = 0.98), and a sister clade relationship between Chinchilloidea and Octodontoidea (Figure 1, BP = 88, PP = 0.92). In spite of these high support values, AU tests indicate that the best alternatives to these arrangements within Caviomorpha cannot be rejected (Table 1, *p*-value = 0.148–0.206). Similarly, analyses using RY coding or removal of third codon positions, as well as protein sequence analysis, support other relationships within Caviomorpha (data not shown). This suggests that additional species sampling is needed in order to robustly solve caviomorph relationships at the superfamily level.

### Solving the base of the rodent tree

The most important unresolved relationship in rodent systematics is the one at the base of the rodent tree. To date, no phylogenetic analysis has been able to resolve this question with strong support, whether based on nucleotide sequence data [24,25,29,31], SINE data [36,44], or morphological data [8,10]. The only exception is the analysis of Montgelard et al. [32], which supports a basal position of the mouse-related clade after removal of fast-evolving nucleotide positions. Our nucleotide-based ML and Bayesian analyses (all three codon positions; Figure 1) place the squirrel-related clade at the base of the rodent tree. Our Bayesian analysis with the data partitioned by gene and partially partitioned by codon position (1^st^- and 2^nd^-position sites combined within genes, 3^rd^-position sites for each gene separate) appears to provide strong support for this relationship (PP > 0.90), but the partitioned ML bootstrap support values are much lower (Table 2, BP = 51). It is possible for Bayesian PP values to be artificially inflated under circumstances of a near-trichotomy [45]. With this single Bayesian analysis being the only suggestion of strong support, and with the corresponding ML bootstrap support being so low, we hesitate to give much weight to the partitioned Bayesian result at the present time.

Recently, Rodriguez-Ezpeleta et al. [33] have shown that the presence of conflicting phylogenetic and non-phylogenetic signal in a dataset may result in weakly supported nodes. They suggested various approaches to remove the non-phylogenetic signal and thus increase the ability to resolve difficult phylogenetic relationships. To evaluate whether the resolution of the basal relationships among rodents could be improved by reducing non-phylogenetic signal in our dataset, we tested all the approaches suggested by Rodriguez-Ezpeleta et al. [33].

#### Character recoding

Third codon-position sites evolve the fastest, and are thus the most likely source of non-phylogenetic signal. In an attempt to reduce the non-phylogenetic signal, we performed analyses using RY coding for these positions. We also explored the extreme solution of removing all third codon-position sites. However, none of the three possible basal branching topologies was highly supported under these alternatives (Table 2). The only signal that can be seen is that a basal position of the mouse-related clade is not supported by the analysis of either the nucleotide dataset with only the first two codon positions, or the protein sequence dataset (Table 2, BP < 1).

#### Removal of fast-evolving positions

**Figure 2:**
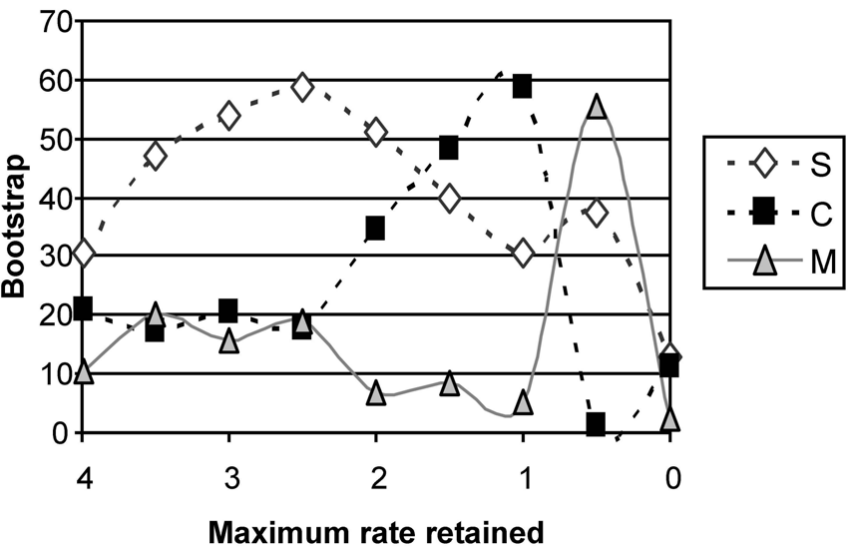
**Bootstrap support as a function of the maximum evolutionary rate of site retained in the data**. S: the best topology with the squirrel-related clade at the base of the rodent tree; M: the best topology with the mouse-related clade at the base of the rodent tree; C: the best topology with Ctenohystrica at the base of the rodent tree. Nine datasets were considered: (1) all sites (6,255 base pairs (bps); rates range from -0.698 to 3.989); (2) sites with rate ≤ 3.5 (6,114 bps); (3) sites with rate ≤ 3.0 (6,058 bps); (4) sites with rate ≤ 2.5 (5,997 bps); (5) sites with rate ≤ 2.0 (5,896 bps); (6) sites with rate ≤ 1.5 (5,759 bps); (7) sites with rate ≤ 1 (5,444 bps); (8) sites with rate ≤ 0.5 (4,997 bps); and (9) sites with rate ≤ 0.0 (4,179 bps).

Nine datasets were delimited by retaining sites based on their inferred site-specific rates: (1) all sites (6,255 base pairs (bps); rates range from -0.698 to 3.989); (2) sites with rate ≤ 3.5 (6,114 bps); (3) sites with rate ≤ 3.0 (6,058 bps); (4) sites with rate ≤ 2.5 (5,997 bps); (5) sites with rate ≤ 2.0 (5,896 bps); (6) sites with rate ≤ 1.5 (5,759 bps); (7) sites with rate ≤ 1 (5,444 bps); (8) sites with rate ≤ 0.5 (4,997 bps); and (9) sites with rate ≤ 0.0 (4,179 bps). The bootstrap support as a function of the maximal evolutionary rate of site retained is presented in Figure 2. Removal of the fastest evolving sites (rate removed ≥ 2.5) improves the support in favor of a basal position of the squirrel-related from 30% to 59% while support for alternative topology remains below 25%. However, no clear trend can be found as bootstrap support remains below 60% in all analyses. It is worth noting that the topology supporting a basal position of the mouse-related clade is again the least supported, except for the dataset with maximum rate ≤ 0.5. We do not believe that this result effectively supports an early divergence of the mouse-related clade, because slight modification of the rate cutoff substantially changes the topology. For example, while the dataset with maximum rate ≤ 0.5 supports a basal position of the mouse-related clade, the dataset with maximum rate ≤ 0.6 supports a basal position of the Ctenohystrica. Note that Montgelard et al. did not study the effect of varying their cut-off value. Finally, the support for all three topologies drops when sites with rate higher than zero are removed, possibly reflecting the fact that only 801 out of 2,858 informative characters remained in this dataset.

**Figure 3:**
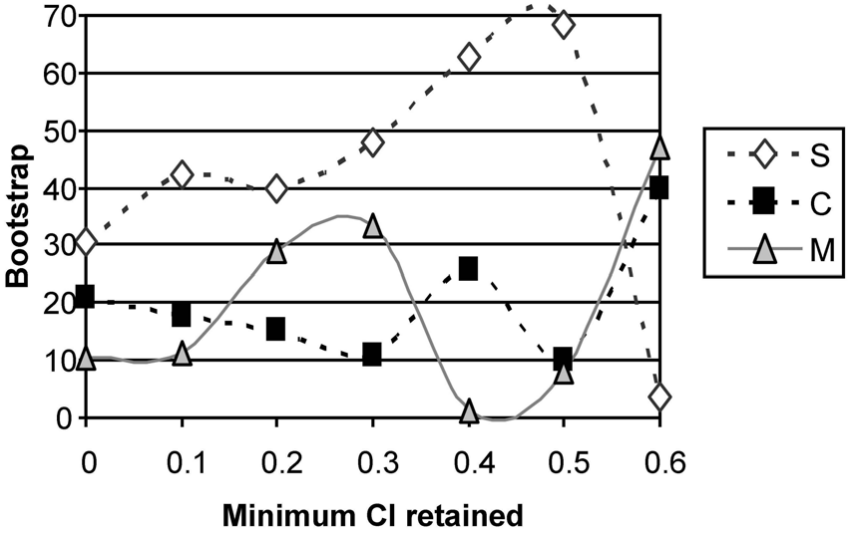
**Bootstrap support as a function of the minimum CI of sites retained in the data**. S: the best topology with the squirrel-related clade at the base of the rodent tree; M: the best topology with the mouse-related clade at the base of the rodent tree; C: the best topology with Ctenohystrica at the base of the rodent tree. Seven datasets were considered: (1) all sites (6,255 bps; CI range 0.0625–1); (2) sites with CI > 0.1 (6,210 bps); (3) sites with CI > 0.2 (5,864 bps); (4) sites with CI > 0.3 (5,432 bps); (5) sites with CI > 0.4 (4,833 bps); (6) sites with CI > 0.5 (4,119 bps); and (7) sites with CI > 0.6 (4,023 bps).

Similarly, seven datasets were considered by retaining sites based on their consistency index (CI): (1) all sites (6,255 bps; CI range 0.0625–1); (2) sites with CI > 0.1 (6,210 bps); (3) sites with CI > 0.2 (5,864 bps); (4) sites with CI > 0.3 (5,432 bps); (5) sites with CI > 0.4 (4,833 bps); (6) sites with CI > 0.5 (4,119 bps); and (7) sites with CI > 0.6 (4,023 bps). When retaining sites according to their maximal CI value (Figure 3), we observe an increase in the bootstrap support in favor of a basal position of the squirrel-related clade from 30.6% to 68.2%, which might suggest that this represents the phylogenetic signal. This support drops when sites with CI ≤ 0.6 are removed, which might come from the fact that only 626 informative characters remain in this dataset.

#### Use of site-heterogenous mixture model

The phylogenetic trees obtained under the CAT model did not help resolving the basal rodent relationships. The CAT analysis suggests that the squirrel-related clade is the first rodent lineage to diverge. However, no strong support in favor of this relationship is found, whether reconstructions are based on nucleotide or protein sequences (PP_DNA _= 0.57; PP_PROTEIN _= 0.75).

#### Use of complex evolutionary models for protein sequences

The use of more complex evolutionary models did not completely solve basal rodent relationships. Again, a basal position of the mouse-related clade is generally the least likely, and this hypothesis is even rejected using AU tests under either the JTT+Γ model or the rate-shift model (Table 3). However, a basal position of the Ctenohystrica cannot be excluded. This finding is in agreement with both the nucleotide analysis based on the first two codon positions and the nucleotide analysis with fast-evolving sites removed.

**Table 3:** Maximum log-likelihood scores and AU test *p*-values under different models of sequence evolution for three possible basal rodent relationships.

**Model of sequence evolution**	**JTT + Γ_4_**		**Rate shift model + Γ_4_**		**Codon model + Γ_4 _(without positive selection)**	
**Topology**	**Diff -ln L**	***p*-value**	**Diff -ln L**	***p*-value**	**Diff -ln L**	***p*-value**

Squirrel-related clade at the base	<32,963.68>	0.580	<32,936.35>	0.586	0.9	0.517
Ctenohystrica at the base	0.2	0.563	0.3	0.558	<77,788.57>	0.623
Mouse related clade at the base	3.2	**0.031**	3.1	**0.035**	4.0	0.158

Significant *p*-values are indicated in bold. The log-likelihood value of the best tree is indicated between brackets.

The nucleotide sequences were also analyzed using codon models. No support for positive selection was found, and hence, we only report the results obtained using the M8a model, which does not allow sites to evolve under positive selection. Under this model, a basal position of the Ctenohystrica is the most likely. However, the fit of the data to this topology is not significantly better than alternative topologies (0.9 and 4.0 log-likelihood point differences, for the topology with a basal position of the squirrel-related clade and the topology with a basal position of the mouse-related clade, respectively). The three possible rootings of the rodent tree are thus not statistically different based on AU tests (Table 3).

## Conclusion

Our phylogenetic reconstructions provide a well-resolved rodent tree, except for a few nodes and the basal relationships among the main rodent clades. Unlike Montgelard et al. [32], removing fast evolving characters did not improve the resolution at the base of the rodent tree. This lack of resolution remained when all the other methods suggested by Rodriguez-Ezpeleta et al. [33] to increase tree resolution were applied. Surprisingly, using the JTT and the rate-shift models, we were able to reject a basal position of the mouse-related clade supported by Montgelard et al. [32] and support instead a basal position of the squirrel-related clade (a topology rejected by Montgelard et al. [32]). This suggests that removing fast evolving positions is not a panacea to solve phylogenetic conflicts, since different datasets can lead to significantly different results when using this approach.

More generally, our results suggest that the low support at the base of the rodent tree cannot be attributed only to the presence of conflicting non-phylogenetic signal, since removing such non-phylogenetic signal failed to significantly increase the tree resolution. We thus hypothesize that this lack of resolution reflects rapid radiation at the base of the rodent tree and possibly incomplete lineage sorting. Indeed, rodents were already highly diversified in the Paleocene and Early Eocene. Many extinct families are identified in these geological periods (i.e., Decipomyidae, Alagomyidae, Ivanantoniidae, Sciuravidae, Ischyromyidae, Theridomorpha, and Yuomyidae). According to recent phylogenetic work based on fossils and extant taxa [8], some of these ancient families are sister clades of extant clades. In particular, Theridomorpha might be related to Sciuroidea, Sciuravidae to the mouse-related clade, and Yuomyidae to the Ctenohystrica [8]. This supports the idea that the divergences among Ctenohystrica, the mouse-related clade, and the squirrel-related clade occurred during the explosive radiation of rodents in the Paleocene.

Our results further suggest that a basal position of the mouse-related clade is the least likely, while a basal position of the squirrel-related clade may be the most likely. Interestingly, structural analysis of B1 retroposon elements also provides additional support in favor of an early divergence of the squirrel-related clade [35,44]. The basal position of the squirrel-related clade may further be supported by the fact that the earliest fossils representative of the Gliridae, Aplodontidae, and Sciuridae families are protrogomorphous, while most early Ctenohystrica and most early representatives of the mouse-related clade are hystricomorphous [see review of character states in [8]]. Consequently, an early divergence of the squirrel-related clade appears to be the most parsimonious evolutionary scenario, given our current knowledge.

## Methods

### Taxon sampling

All suborders and super families of the order Rodentia listed by Carleton and Musser [46] are included in the analysis (Additional file 1). The tree was rooted with the closest rodent outgroups: representative lagomorphs, representative primates, Cynocephalus (the flying lemur, order Dermoptera), and Tupaia (tree shrew, order Scandentia). Rodents together with lagomorphs, primates, flying lemurs, and tree shrews form a clade called Euarchontoglires or Supraprimates [21,47-49].

### DNA amplification and sequencing

Ethanol-preserved samples, frozen tissue samples, or previously purified genomic DNAs were obtained from the donor institutions listed in Additional file 2. Total DNA was extracted according to Sambrook, Fritsch, and Maniatis [50] with slight modifications. Fragments from the following six nuclear genes were sequenced: the alpha 2B adrenergic receptor (ADRA2B); the cannabinoid receptor 1 (CB1); the growth hormone receptor (GHR); the interphotoreceptor retinoid binding protein (IRBP); the recombination activating gene 2 (RAG2); and the von Willebrand factor (vWF). These nuclear genes were chosen for the following reasons: (i) a large number of sequences are already available for those genes, especially within rodents; (ii) these genes have been shown to contain phylogenetic information within rodents and between mammalian orders [21,26,30]; (iii) these genes are not genetically linked to one another (their location is variable, on chromosomes 2, 4, 15, 14, 2, and 6 in Mus, chromosomes 3, 5, 2, 16, 3, and 4 in Rattus, and chromosomes 2, 6, 5, 10, 11, and 12 in Homo); and (iv) no interactions among these proteins were previously reported.

Amplification of ADRA2B, IRBP, and vWF was performed as described in Huchon et al [29]. Amplification of CB1 was performed in two steps. A first amplification was performed with primers CB1-D1: 5'-GGCTCAAATGACATTCAGTACGAA-3' and CB1-R1: 5'-GAGTCCCCCATGCTGTTATCTAGAGGCTG-3', followed by a re-amplification of the initial PCR product using primers CB1-D2: 5'-CAGTACGAAGATATCAAAGGAGACATGGC-3' and CB1-R2: 5'-GAGTCCCCCATGCTGTTATCTAGAGGCTG-3'. Amplification of RAG2 and GHR was performed similarly. For RAG2, the first amplification was performed with primers RAG2-D1 5'-CGCTGCACAGAGAAAGACTT-3' and RAG2-R1: 5'-AAGGATTTCTTGGCAGGAGT-3', followed by a re-amplification of the initial PCR product using primers RAG2-D2: 5'-TAYAGYCGAGGGAAAAGYATGGG-3' and RAG2-R2: 5'-GACAAGTGGATGAGTGTGCGTTC-3'. For GHR the first amplification was performed with primers GHR-D1 5'-TAGGAAGGAAAATTRGARGARGTNAA-3'and GHR-R1: 5'-AAGGCTANGGCATGATRTTRTT-3', followed by a re-amplification of the initial PCR product using primers GHR-D2: 5'-GGAAAATTRGAGGAGGTGAAYACNATHTT-3' and GHR-R2: 5'-GATTTTGTTCAGTTGGTCRGTRCTNAC-3' or GHR-R1. Purification of the PCR products and sequencing were performed according to Huchon et al. [29]. Sequence accession numbers are available in Additional file 1.

### Sequence alignment

DNA sequences were translated and the corresponding protein sequences were aligned using both PROBCONS [51] and MAFFT [52]. PROBCONS alignments were conducted with three consistency steps and 500 iterative refinement repetitions. MAFFT alignments were conducted with the L-INS-i option. The positions that differed between both alignments were removed using SOAP [53]. The DNA alignments were then computed based on the protein alignments using the program PAL2NAL [54]. The number of DNA positions included in each gene partition after using SOAP is indicated in Table 4. The DNA and protein sequence alignments are provided in Additional file 3 and Additional file 4, respectively.

**Table 4:** Number of positions in each gene partition.

	**Constant**	**Variable uninformative**	**Informative**	**Total**
A2AB	500	98	464	1,062

CB1	694	70	319	1,083

GHR	219	103	470	792

IRBP	440	172	627	1,239

RAG2	430	111	365	906

vWF	388	172	613	1,173

Total	2,671	726	2,858	6,255

### ML analyses of the concatenated dataset

Phylogenetic tree reconstructions were performed on the concatenated nucleotide dataset using the ML criterion. The program MODELTEST 3.07 [55] was used to determine the best probabilistic model of DNA sequence evolution using the Akaike Information Criterion. The best model was found to be GTR+Γ+I. ML searches for the best trees were performed using the program PAUP* [56]. The parameters of the model and the ML tree were then determined by successive approximation [57]. The initial parameter values were those estimated by MODELTEST 3.07, and those values were used for a first round of heuristic search starting with a Neighbor-Joining (NJ) tree and using TBR branch-swapping. Parameters were then estimated on the resulting tree and used for another round of heuristic search. The process was repeated until all parameter values were stable. Bootstrap percentages were estimated from 100 pseudo-replicates using the best estimated parameters, a NJ starting tree, and TBR branch-swapping.

Phylogenetic trees were also reconstructed based on protein sequences. The protein sequence alignment is provided in Additional file 4. The program PROTTEST 1.3 [58] was used to estimate the best model of protein sequence evolution. The best model was found to be JTT+Γ+I. Phylogenetic trees were then reconstructed with the program PHYML [59] using the ML model identified by PROTTEST.

### ML analyses of the partitioned dataset

Three different partitioned ML-analyses were conducted on the nucleotide dataset with RAxML [60]. The first analysis considered each gene as an independent partition (six partitions). The second analysis considered each codon position as an independent partition (three partitions). The third analysis considered each codon position of each gene as an independent partition (18 partitions). The GTR+Γ+I model was applied to all partitions, individual α-shape parameters, substitution rates, and base frequencies were estimated and optimized separately for each partition. Bootstrap support was estimated using 100 pseudo-replicates.

### Bayesian analyses

Bayesian analyses were performed on the nucleotide dataset using the program MrBayes v3.1.2 [61]. Prior distributions for parameters in the Bayesian analyses were: topology, uniform; branch lengths, exponential (λ = 10); alpha parameter of the Γ distribution, uniform (0.05,50.0); *p*_inv_, uniform (0,1); κ, beta (1.0,1.0); R-matrix, Dirichlet (1,1,1,1,1,1); base frequencies, Dirichlet (1,1,1,1). Each run included two independently started chains, each beginning with a different, randomly chosen tree. From each starting tree, four related MCMC chains (one cold and three incrementally heated) were run. The temperature parameter λ was set to produce chain-swap frequencies in the range of 10–30%. Posterior distribution estimates were based on sampling the cold chain every 250 generations. Initial runs were allowed to stop at 10^7 ^cycles if the percent standard deviation among bifurcation split probabilities for the two separate chains was less than 0.01. In those cases, the first 5 × 10^6 ^cycles were discarded as burn-in. Additional longer runs were performed if needed, with the first 50% of samples discarded as burn-in. The dataset was divided into 12 partitions, two for each gene. For each gene, the first- and second-position sites were combined into a single partition, and the third-position sites in a separate partition. Analyses were conducted with each partition assigned either the HKY85+Γ or the GTR+Γ model, with the exception of the 1^st^- plus 2^nd^-position site partition for CB1, which used either HKY85+I or GTR+I, because of the extremely low number of variable sites in that partition. The GTR models were preferred by Bayes' Factors, while the HKY85 models were favored by the Bayesian information criterion (BIC). However, both models gave very similar results, as did a separate set of analyses with the CB1 1^st^- plus 2^nd^-position sites excluded.

Bayesian analyses under the CAT +Γ_4 _model were performed using the program Phylobayes 2.1c [34,62]. Both for the DNA and protein datasets, two chains were run for 100,000 cycles and trees were sampled every 100 cycles after the first 25,000 cycles. As recommended in Phylobayes, the maximum difference in bipartition frequencies between the two chains was below 0.1, indicating a "good run" (for DNA, maxdiff = 0.051; for protein, maxdiff = 0.044). The phylogenetic trees obtained under the CAT +Γ_4 _model are available in Additional file 5.

### Testing alternative hypotheses

The ML tree was compared to several constrained topologies using various likelihood-based tests as implemented in the program CONSEL v0.1i [63]. Eleven alternative topologies were considered. 1 – The best tree placing the Ctenohystrica at the base of Rodentia. 2 – The best tree placing the mouse-related clade at the base of Rodentia. 3 – The best tree placing Myodonta at the base of the mouse-related clade. 4 – The best tree placing Anomaluromorpha at the base of the mouse-related clade. 5 – The best alternative that does not support monophyly of Chinchilloidea+Octodontoidea. 6 – The best alternative that does not support monophyly of Cavioioidea+Erethizontoidea. 7 – The best tree placing Phiomorpha at the base of the Hystricognathi. 8 – The best tree placing Caviomorpha at the base of the Hystricognathi. 9 – The best alternative that does not support monophyly of the mouse-related clade. 10 – The best alternative that does not support monophyly of the squirrel-related clade. 11 – The best alternative that does not support monophyly of the Ctenohystrica. The best alternatives were built using constrained ML heuristic searches. Each search was conducted starting with an NJ tree, using the TBR branch-swapping option, and the parameters of the unconstrained ML tree. Site-wise log-likelihoods were computed with PAUP* using the parameters of the best ML tree.

### Removal of fast-evolving positions

Following the approach of Rodriguez-Ezpeleta et al. [33], fast evolving sites were determined according to their site-wise rates calculated with the program Rate4Site [64] using the Tamura-Nei substitution model [65] with 16 discrete rate categories used to approximate the Gamma distribution. Rates were computed for three topologies: the ML tree topology obtained as describe above (i.e., the topology with the squirrel-related clade at the base), and the two alternative topologies (the mouse-related clade at the base and the Ctenohystrica at the base). All other nodes were identical between the topologies. Nucleotide sites were classified according to their average rate over the three topologies. Rates were normalized so that the average rate across all sites was 0. Rates ranged between – 0.698 to 3.989. The fastest-evolving sites were then progressively removed to create nine datasets: all sites; sites with rates ≤ 3.5; sites with rates ≤ 3.0; sites with rates ≤ 2.5; sites with rates ≤ 2.0; sites with rates ≤ 1.5; sites with rates ≤ 1.0; sites with rates ≤ 0.5; and sites with rates ≤ 0. Bootstrap values were computed with the program Treefinder [66]. For each dataset, the ML tree and parameters were estimated by Treefinder under the GTR+Γ+I model. These ML parameters were then used to perform a bootstrap analysis using 500 replicates.

Removing sites based on their evolutionary rate does not allow differentiation between sites with few character states (e.g. sites with only purines or only pyrimidines) from sites with all possible character states (e.g. sites with all four bases). However, if two positions evolve under the same substitution rate, we can expect the one with more character states to be less homoplasious. Consequently, we used CI values as a measure of the level of homoplasy. It is worth noting that CI and site specific rates are weekly correlated (Additional file 6). For each of the three topologies described above, the CI of each site was computed using PAUP* and sites were classified according to their average CI values across the three topologies. Seven datasets were constructed by eliminating some sites based on their CI values (retaining all sites; retaining sites with CI > 0.1; retaining sites with CI > 0.2; retaining sites with CI > 0.3; retaining sites with CI > 0.4; retaining sites with CI > 0.5; and retaining sites with CI > 0.6). Bootstrap values were then computed as described above.

### Character recoding

Since the third codon position generally evolves at the highest rate, we performed two types of ML analyses that were intended to reduce the impact of this high rate. Phylogenetic trees were reconstructed either without third codon position or using RY coding at the third codon position. The program MODELTEST 3.07 was used to infer the model of sequence evolution and phylogenetic trees were reconstructed using PAUP* as described above.

### Use of complex evolutionary models

It has been shown that using codon-based models can improve phylogenetic inference from protein-coding genes [67]. This method is computationally intensive, thus precluding the computation of bootstrap values for a dataset of 49 species. To this end, maximal log-likelihoods of the three topologies involving basal rodent relationships were compared using the program CONSEL [63]. For this comparison, site-wise log-likelihoods of each topology under two codon models were computed using the program SELECTON version 2.3 [68]. The first model, M8, allows for positive selection operating on the protein [69]. The second model, M8a, does not allow for positive selection [70]. In both codon analyses, four Gamma-rate categories were used to account for among-site rate variation.

Heterotachy (covarion) is define as the variation of the evolutionary rate within a given site, and it was shown to have an impact on phylogenetic inferences [e.g., [71]]. Consequently, the covarion model developed by Galtier [72], extended to amino-acids [73] was used to compute the site-wise log-likelihoods of the three topologies involving basal rodent relationships. Four Gamma-rate categories were used to account for among-site rate variation. The C++ code for computing site-wise log-likelihoods under the covarion model is available from the authors upon request.

The support for each topology under the codon and covarion models was compared to the support under the JTT model. The program Rate4Site was used to obtain the site-wise log-likelihood of the three topologies tested, and these site-wise log-likelihoods were used as input to CONSEL.

## Authors' contributions

SBK and HM carried out the sequencing and participated in the sequence alignment and analyses. OP conducted the phylogenetic analysis based on complex evolutionary models. TP supervised the analysis based on complex evolutionary models. RWD and DH conceived of the study, participated in its design and coordination, and wrote the manuscript. All authors read and approved the final manuscript.

## Supplementary Material

Additional file 1 **Accession number table**. List of all specimens and accession numbers for each gene sequenced. Click here for file

Additional file 2 **Tissue samples table**. Origin of the tissue samples considered and genes amplified. Click here for file

Additional file 3 **DNA sequence alignment**. Concatenated DNA sequence alignment (in Nexus format) used to reconstruct the phylogenetic tree present in figure 1. Click here for file

Additional file 4 **Protein sequence alignment**. Concatenated protein sequence alignment (in Nexus format) used in the phylogenetic analyses. Click here for file

Additional file 5 **Phylogeny reconstructed under the CAT model**. Figure of the phylogenetic trees obtained under the Bayesian CAT model. Click here for file

Additional file 6 **Variation of the CI value as a function of the site specific evolutionary rate**. Scatterplot visualizing the variation of the CI value as a function of the site specific evolutionary rate. Click here for file
