# Supplementary material for: Rodent phylogeny revised: analysis of six nuclear genes from all major rodent clades
Source: BMC Evol Biol. 2009 Apr 2;9:71. doi: 10.1186/1471-2148-9-71 (PMC2674048; doi:10.1186/1471-2148-9-71)
Supplement: Additional file 1 — Accession number table. List of all specimens and accession numbers for each gene sequenced. [file 1471-2148-9-71-S1.doc]

### Additional file 1 – List of all specimens and accession nunbers for each gene sequenced

|  | **Species** | **ADRA2B** | **IRBP** | **vWF** | **GHR** | **RAG2** | **CB1** |
| --- | --- | --- | --- | --- | --- | --- | --- |
| ***Rodentia*** |  |  |  |  |  |  |  |
| **Sciurognathi** |  |  |  |  |  |  |  |
| Muridae | *Mus musculus* | M94583 | AF126968 | AJ238390 | U49266 | NM_009020 | BC070447 |
|  | *Rattus norvegicus* | M32061 | AJ429134 | AJ224673 | X16726 | AY303209 | U40395 |
|  | *Mesocricetus auratus* | FM162044* | FM162052* | FM162063* | FM162069* | FM162083* | FM162115* |
|  | *Tachyoryctes sp.* | AJ427264 | AJ427231 | AJ402713 | FM162070* | FM162084* | FM162116* |
|  | *Spalax sp. (1)* | AM407905 | U48589 | FM162064* | AY294898 | FM162085* | FM162117* |
|  | *Rhizomys pruinosus* | X | AF297283 | X | AY294899 | AY303210 | AY303191 |
|  | *Acomys russatus* | FM162045* | FM162053* | FM162065* | FM162071* | FM162086* | FM162118* |
|  | *Gerbillus dasyurus* | FM162046* | FM162054* | FM162066* | FM162072* | FM162087* | FM162119* |
|  | *Microtus socialis* | FM162047* | FM162055* | FM162067* | FM162073* | FM162088* | FM162120* |
| Dipodidae | *Dipus sagitta* | AJ427263 | AJ427232 | AJ224665 | AM407908 | AY303203 | AY303184 |
|  | *Jaculus jaculus* | AM407906 | AM407907 | AJ297765 | AF332040 | FM162089* | FM162121* |
| Heteromyidae | *Dipodomys merriami* | AJ427261 | AJ427233 | AJ427226 | FM162074* | FM162090* | FM162122* |
| Geomyidae | *Thomomys sp.* (2) | AJ427262 | AJ427234 | AJ427227 | AM407911 | AY303215 | AY303196 |
|  | *Geomys breviceps* | AM407913 | AM407914 | AM407915 | FM162075* | FM162091* | FM162123* |
| Gliridae | *Eliomys quercinus* | FM162048* | FM162056* | FM162068* | FM162076* | FM162092* | FM162124* |
|  | *Glis glis* | AJ427258 | AJ427235 | AJ224668 | FM162077* | FM162093* | FM162125* |
|  | *Dryomys sp.* (2) | AJ427257 | AJ427236 | AJ224666 | AY294896 | AY303204 | AY303185 |
| Sciuridae | *Marmota monax* | AJ427255 | AJ427237 | AJ224671 | AM407917 | FM162094* | FM162126* |
|  | *Sciurus aestuans* | FM162049* | FM162057* | AM407918 | FM162078* | FM162095* | FM162127* |
| Aplodontidae | *Aplodontia rufa* | AJ427256 | AJ427238 | AJ224662 | AF332030 | AY303198 | AY303179 |
| Castoridae | *Castor canadensis* | AJ427260 | AJ427239 | AJ427228 | AF332026 | AY303199 | AY303180 |
| Anomaluridae | *Anomalurus sp.* | AJ427259 | AJ427230 | AJ427229 | AM407919 | FM162096* | FM162128* |
| Pedetidae | *Pedetes sp.* (4) | AM407920 | AJ427241 | AJ238389 | AF332025 | AY303208 | AY303189 |
| Ctenodactylidae | *Massoutiera mzabi* | AJ427265 | AJ427242 | AJ238388 | AM407921 | X | FM162129* |
|  | *Ctenodactylus sp.* (5*)* | AM407922 | AM407923 | AJ238387 | AF332042 | FM162097* | FM162130* |

|  | **Species** | **ADRA2B** | **IRBP** | **vWF** | **GHR** | **RAG2** | **CB1** |
| --- | --- | --- | --- | --- | --- | --- | --- |
| **Hystricognathi** |  |  |  |  |  |  |  |
| Thryonomyidae | *Thryonomys swinderianus* | AJ427267 | AJ427243 | AJ224674 | AF332035 | FM162098* | FM162131* |
| Petromuridae | *Petromus typicus* | AJ427268 | AJ427244 | AJ251144 | FM162079* | FM162099* | FM162132* |
| Bathyergidae | *Bathyergus suillus* | AJ427252 | AJ427251 | AJ238384 | FM162080* | FM162100* | FM162133* |
|  | *Heterocephalus glaber* | AM407924 | AM407925 | AJ251134 | AF332034 | FM162101* | FM162134* |
| Hystricidae | *Trichys fasciculata* | AJ427266 | AJ427245 | AJ224675 | FM162081* | FM162102* | FM162135* |
|  | *Hystrix africaeaustralis* | X | FM162058* | X | AF332033 | FM162103* | FM162136* |
| Chinchillidae | *Chinchilla lanigera* | AJ427271 | AJ427246 | AJ238385 | AF332036 | AY303201 | AY303182 |
| Dinomyidae | *Dinomys branickii* | AM050859 | AM050862 | AJ251145 | AF332038 | FM162104* | FM162137* |
| Echimyidae | *Echimys chrysurus* | AJ427269 | AJ427247 | AJ251141 | FM162082* | FM162105* | FM162138* |
| Abrocomyidae | *Abrocoma sp (6)* | FM162050* | FM162059* | AJ251143 | AF520643 | FM162106* | FM162139* |
| Capromyidae | *Capromys pilorides* | AM407926 | AM407927 | AJ251142 | AF433949 | FM162107* | FM162140* |
| Octodontidae | *Octodon sp. (*7) | AM050860 | AM050863 | AJ238386 | AM407928 | FM162108* | FM162141* |
| Caviidae | *Cavia porcellus* | AJ271336 | AJ427248 | AJ224663 | AF238492 | NCBI trace data | DQ355990 |
|  | *Hydrochaeris hydrochaeris* | FM162051* | FM162060* | AJ251137 | AF433948 | FM162109* | FM162142* |
| Agoutidae | *Agouti paca* | AM050861 | AM050864 | AJ251136 | AF433928 | FM162110* | FM162143* |
| Erethizontidae | *Erethizon dorsatum* | AJ427270 | AJ427249 | AJ251135 | AF332037 | AY303205 | AY303186 |
| **Lagomorpha** |  |  |  |  |  |  |  |
| Leporidae | *Oryctolagus cuniculus* | Y15946 | Z11812 | U31618 | NM001082636 | FM162111* | AY303190 |
|  | *Lepus sp.* (8) | AJ427254 | AJ427250 | AJ224669 | AF332016 | FM162112* | FM162144* |
| Ochotonidae | *Ochotona princeps* | AJ427253 | AY057832 | AJ224672 | AF332015 | AY303207 | AY303188 |
| **Primates** |  |  |  |  |  |  |  |
| Hominidae | *Homo sapiens* | M34041 | J05253 | M25851 | X06562 | M94633 | U73304 |
| Cercopithecidae | *Macaca mulatta* | AM050852 | AJ313476 | AJ410302 | NM001042667 | XM001114148 | NM001032825 |
| Lemuridae | *Lemur sp. (9)* | AJ891067 | AJ313470 | AJ410292 | AF540627 | AY011956 | AY011595 |
| **Dermoptera** |  |  |  |  |  |  |  |
| Cynocephalidae | *Cynocephalus sp.* (10) | AJ251182 | FM162061* | U31606 | AF540625 | FM162113* | FM162145* |
| **Scandentia** |  |  |  |  |  |  |  |
| Tupaiidae | *Tupaia sp.* (11) | AY150333 | FM162062* | AF061063 | AF332018 | FM162114* | FM162146* |

*FM162044-FM162146: newly determined sequences.

X: gene regions where sequences were not obtained.

1. *Spalax ehrenbergi (*ADRA2B*,* vWF, GHR, RAG2, CB1) and *Spalax zemni*(IRBP*)*
2. *Thomomys talpoides* (ADRA2B, IRBP, vWF) and *Thomomys bottae* (GHR, RAG2, CB1)
3. *Dryomys nitedula* (ADRA2B, IRBP, vWF, RAG2, CB1) and *Dryomys* sp. (GHR).
4. *Pedetes surdaster* (IRBP, vWF) and *Pedetes capensis* (ADRA2B, GHR, RAG2, CB1).
5. *Ctenodactylus vali* (IRBP, vWF) , *Ctenodactylus gundi* (ADRA2B, GHR, RAG2, CB1 ).
6. *Abrocoma bennettii(*ADRA2B, vWF) and *Abrocoma cinerea (IRBP,GHR*, RAG2, CB1).
7. *Octodon lunatus* (ADRA2B, IRBP, vWF) and *Octodon degus* (GHR,RAG2, CB1).
8. *Lepus crawshayi* (ADRA2B, IRBP, vWF), *Lepus capensis* (GHR) and *Lepus starcki* (RAG2, CB1).
9. *Lemur catta* (ADRA2B, IRBP, vWF, RAG2, CB1) and *Eulemur coronatus* (GHR).
10. *Cynocephalus variegates (*ADRA2B, vWF, GHR) and Cynocephalus sp. ( IRBP, RAG2, CB1).
11. *Tupaia belangeri* (ADRA2B, GHR), *Tupaia glis* (vWF) and *Tupaia sp.(* IRBP,RAG2, CB1).
