# Supplementary material for: Rodent phylogeny revised: analysis of six nuclear genes from all major rodent clades
Source: BMC Evol Biol. 2009 Apr 2;9:71. doi: 10.1186/1471-2148-9-71 (PMC2674048; doi:10.1186/1471-2148-9-71)
Supplement: Additional file 2 — Tissue samples table. Origin of the tissue samples considered and genes amplified. [file 1471-2148-9-71-S2.doc]

### Table S2. Origin of the tissue samples considered and genes amplified

| **Tissue number** | **Species** | **Holding university** | **Sampling origin** | **Donor/collector** | **Gene sequenced** |
| --- | --- | --- | --- | --- | --- |
| DH X-046 | *Mesocricetus auratus* | Tel-Aviv University, Israel | ___ | ___ | ADRA2B, vWF, GHR |
| 50643 | *Mesocricetus auratus* | University of Alaska Museum | ___ | ___ | IRBP, RAG2, CB1 |
| T-1703 | *Tachyoryctes* sp. | University  Montpellier II, France | Buhanza, Burundi. | V. Volobouev /  E. Pelé | GHR, RAG2, CB1 |
| DH X-039 | *Spalax erhenbergi* | Tel-Aviv University, colony, Israel | Israel | J. Terkel | vWF |
| ___ | *Spalax erhenbergi* | University of California, Riverside | ___ | M.S. Springer | RAG2, CB1 |
| DH X-034 | *Acomys russatus* | Tel-Aviv University, colony, Israel | Israel | N. Kronfeld-Schor | ADRA2B, vWF, GHR, IRBP, RAG2, CB1 |
| DH X-035 | *Gerbillus dasyurus* | I. Meier Segals Garden for Zoological Research (Tel-Aviv University, Israel) | Israel | D. Eilam | ADRA2B, vWF, GHR, IRBP, RAG2, CB1 |
| DH X-036 | *Microtus socialis* | I. Meier Segals Garden for Zoological Research (Tel-Aviv University, Israel) | Israel | D. Eilam | ADRA2B, vWF, GHR, IRBP, RAG2, CB1 |
| DH X-040 | *Jaculus jaculus* | I. Meier Segals Garden for Zoological Research (Tel-Aviv University, Israel) | Israel | D. Eilam | RAG2, CB1 |
| T-1900 | *Dipodomys merriami* | University of Nevada | Flanigan, Nevada | J. Hayes /  M. R. Banta | GHR, RAG2, CB1 |
| M-1889 | *Geomys breviceps* | Louisiana State University Museum of Natural Science | Vermon Parish, Louisiana, USA | D. L. Dittmann /  D.S. Semple | GHR, RAG2, CB1 |
| T-2166 | *Eliomys quercinus* | University  Montpellier II, France | Navarredonda, Catalonia, Spain | T. Arrizabalaga | ADRA2B, vWF, GHR, IRBP, RAG2, CB1 |
| T-1719 | *Glis glis* | University  Montpellier II, France | Puéchabon, Hérault, France | P. Perret | GHR, RAG2, CB1 |

**Table S2.** Continued

| **Tissue number** | **Species** | **Holding university** | **Sampling origin** | **Donor/collector** | **Gene sequenced** |
| --- | --- | --- | --- | --- | --- |
| 22083 | *Marmota monax* | University of Alaska Museum | Fairbanks, Alaska | ___ | RAG2, CB1 |
| T-1758 | *Sciurus aestuans* | University  Montpellier II, France | Petit saut, French Guyana | T. Arrizabalaga | ADRA2B, GHR, IRBP, RAG2, CB1 |
| H6194 | *Anomalurus sp.* | Texas Cooperative Wildlife Collection | Agumatsa River, Volta Region, Ghana | ___ | RAG2, CB1 |
| T-0390 | *Massoutiera mzabi* | Colony in Lyon, France | Mzabe, Algeria | P. Gouat | CB1 |
| H2206 | *Ctenodactylus gundi* | Texas Cooperative Wildlife Collection | ___ | D. Schlitter | RAG2, CB1 |
| T-0809 | *Thryonomys swinderianus* | University  Montpellier II, France | Koubatchi, Congo | L. Granjon | RAG2, CB1 |
| DH X-005 | *Petromus typicus* | Zoological Society of Philadelphia | ___ | J. Trupkiewicz and R. Hoyt | GHR, RAG2, CB1 |
| T-1847 | *Bathyergus suillus* | ___ | South Africa | C. G. Faulkes/  J. Jarvis | GHR, RAG2, CB1 |
| H819 | *Heterocephalus glaber* | Texas Cooperative Wildlife Collection | ___ | ___ | RAG2, CB1 |
| T-0726 | *Trichys fasciculata* | University  Montpellier II, France | Sabah, Borneo | R. Stuebing | GHR, RAG2, CB1 |
| SP7702 | *Hystrix africaeaustralis* | Texas Cooperative Wildlife Collection | Cape Province, South Africa | ___ | IRBP, RAG2, CB1 |
| DH X-007 | *Dinomys branickii* | Cleveland Metroparks | ___ | C.J. Bonar | RAG2, CB1 |
| A-0024 | *Echimys chrysurus* | University  Montpellier II, France | Petit saut, French Guyana | F. Catzeflis | GHR, RAG2, CB1 |
| NK 30665 | *Abrocoma cinerea* | Museum of Southwestern Biology | ___ | ___ | ADRA2B, IRBP, RAG2, CB1 |
| T-2120 | *Capromys pilorides* | Rotterdam Zoo, Netherland | ___ | M. Mensink | RAG2, CB1 |

**Table S2.** Continued

| **Tissue number** | **Species** | **Holding university** | **Sampling origin** | **Donor/collector** | **Gene sequenced** |
| --- | --- | --- | --- | --- | --- |
| DH X-045 | *Octodon degus* | Tel-Aviv University, Israel | ___ | ___ | RAG2, CB1 |
| 56081 | *Hydrochaeris hydrochaeris* | Texas Cooperative Wildlife Collection | ___ | ___ | ADRA2B, IRBP, RAG2, CB1 |
| T-1555 | *Agouti paca* | University  Montpellier II, France | Petit saut, French Guyana | J.-C. Vié | RAG2, CB1 |
| ___ | *Oryctolagus cuniculus* | University of Cincinnati | ___ | M. Bebehani | RAG2 |
| T-1593 | *Lepus starcki* | University  Montpellier II, France | Entoto, Addis Abeba, Ethiopia | M. Corti | RAG2, CB1 |
| ___ | *Cynocephalus sp.* | University of California, Riverside |  | M.S. Springer | IRBP, RAG2, CB1 |
| ___ | *Tupaia sp.* | University of California, Riverside |  | M.S. Springer | IRBP, RAG2, CB1 |
