# Supplementary material for: Rodent phylogeny revised: analysis of six nuclear genes from all major rodent clades
Source: BMC Evol Biol. 2009 Apr 2;9:71. doi: 10.1186/1471-2148-9-71 (PMC2674048; doi:10.1186/1471-2148-9-71)
Supplement: Additional file 5 — Phylogeny reconstructed under the CAT model. Figure of the phylogenetic trees obtained under the Bayesian CAT model. [file 1471-2148-9-71-S5.doc]

### Additional file 5 – Phylogenetic trees obtained with the CAT model


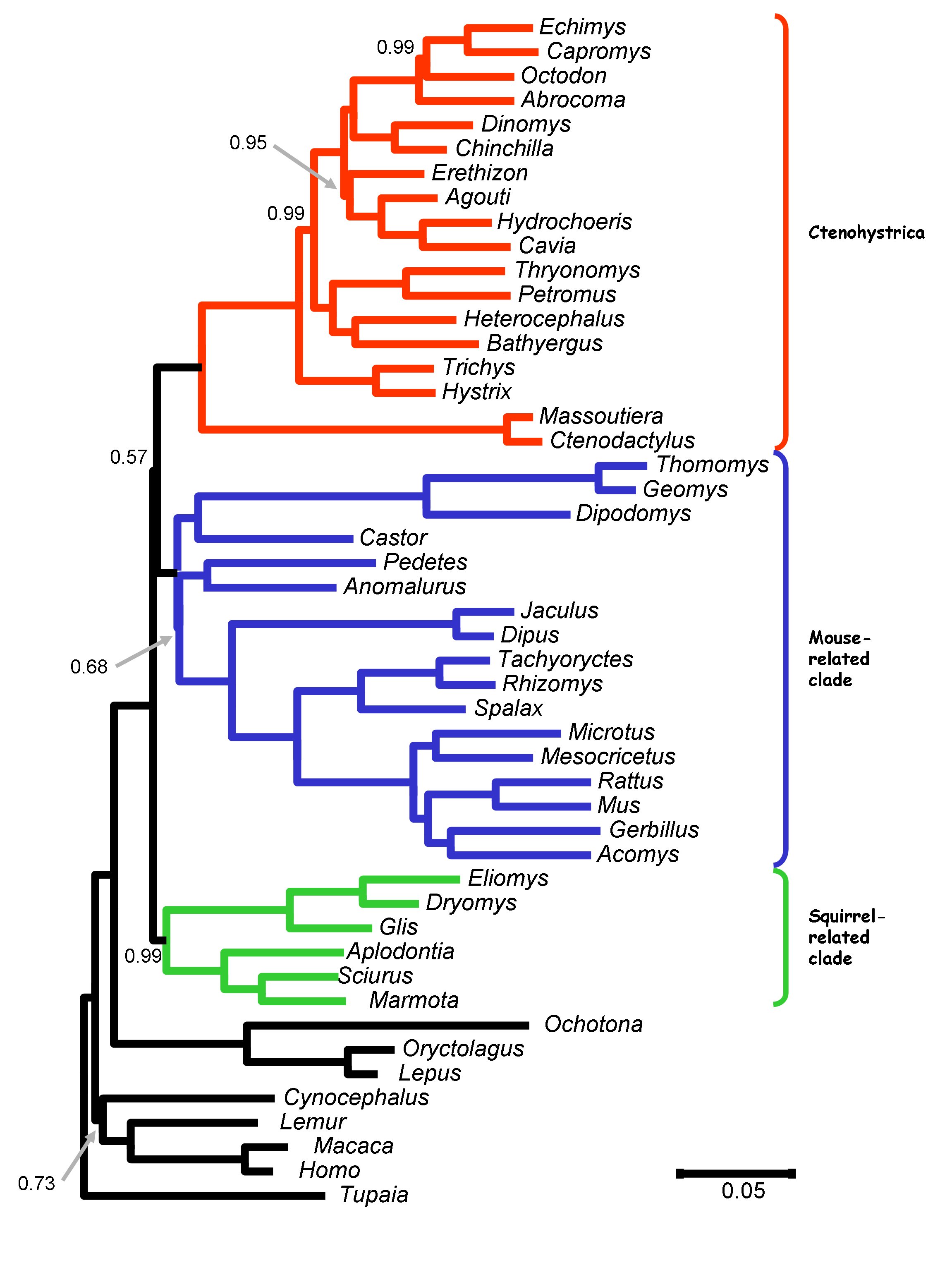


**Bayesian tree obtained for the concatenated DNA data set under the CAT +Γ4 model of sequence evolution.**

All Bayesian posterior probabilities PP = 1.0, unless indicated otherwise near the corresponding nodes.


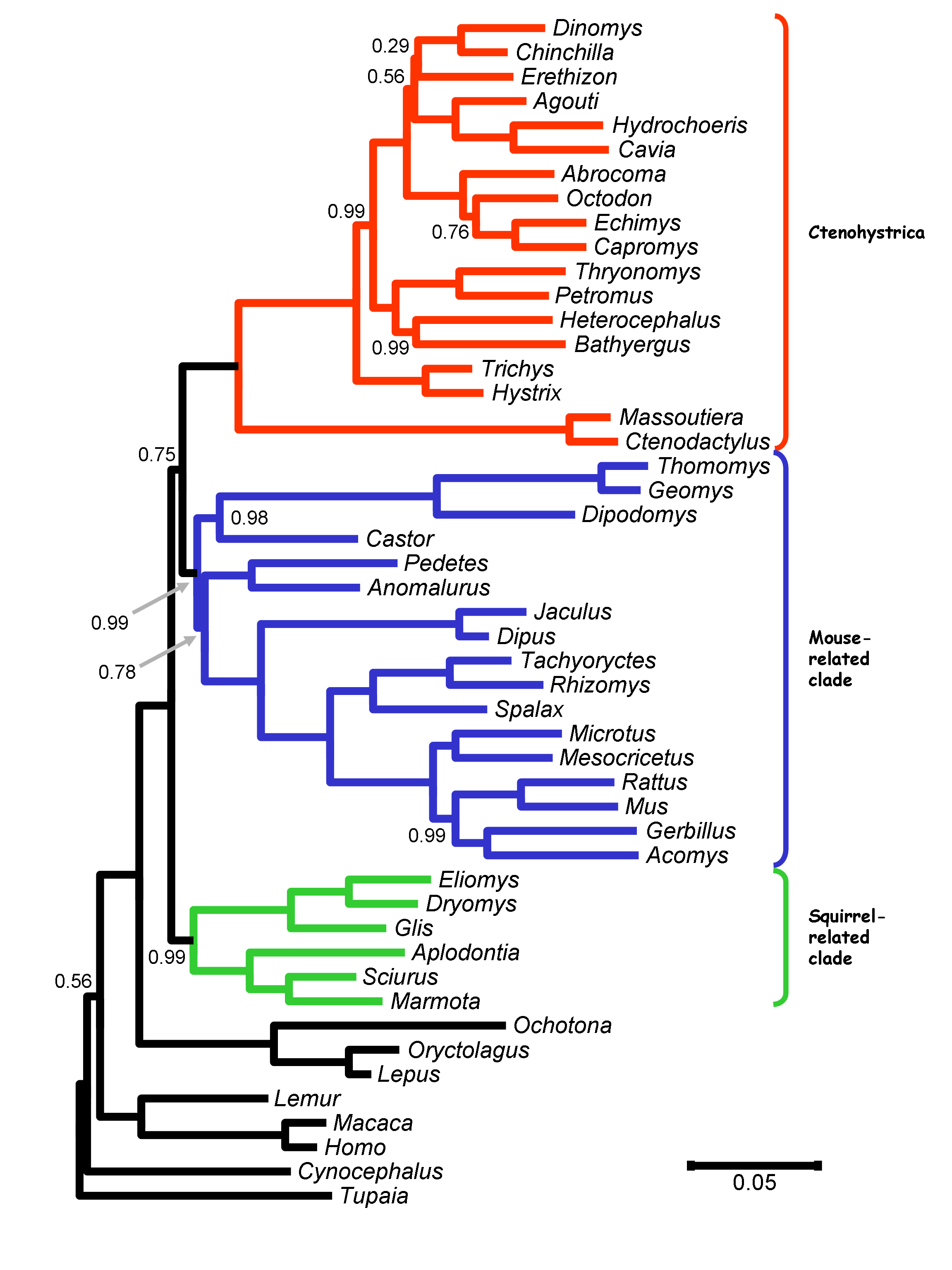


**Bayesian tree obtained for the concatenated protein data set under the CAT +Γ4 model of sequence evolution.**

All Bayesian posterior probabilities PP = 1.0, unless indicated otherwise near the corresponding nodes.
