# Supplementary material for: Rodent phylogeny revised: analysis of six nuclear genes from all major rodent clades
Source: BMC Evol Biol. 2009 Apr 2;9:71. doi: 10.1186/1471-2148-9-71 (PMC2674048; doi:10.1186/1471-2148-9-71)
Supplement: Additional file 6 — Variation of the CI value as a function of the site specific evolutionary rate. Scatterplot visualizing the variation of the CI value as a function of the site specific evolutionary rate. [file 1471-2148-9-71-S6.doc]

### Additional file 6 – Variation of the CI as a function of the site specific evolutionary rate.

It is worth noting that positions with the same CI can exhibit a large range of rates, in particular positions with low CI.
